# Supplementary material for: Enhanced hybridization-proximity labeling discovers protein interactomes of single RNA molecules
Source: Nat Commun. 2025 Oct 20;16:9257. doi: 10.1038/s41467-025-64282-5 (PMC12537909; doi:10.1038/s41467-025-64282-5)
Supplement: Supplementary file 1 — Supplementary Information [file 41467_2025_64282_MOESM1_ESM.pdf]

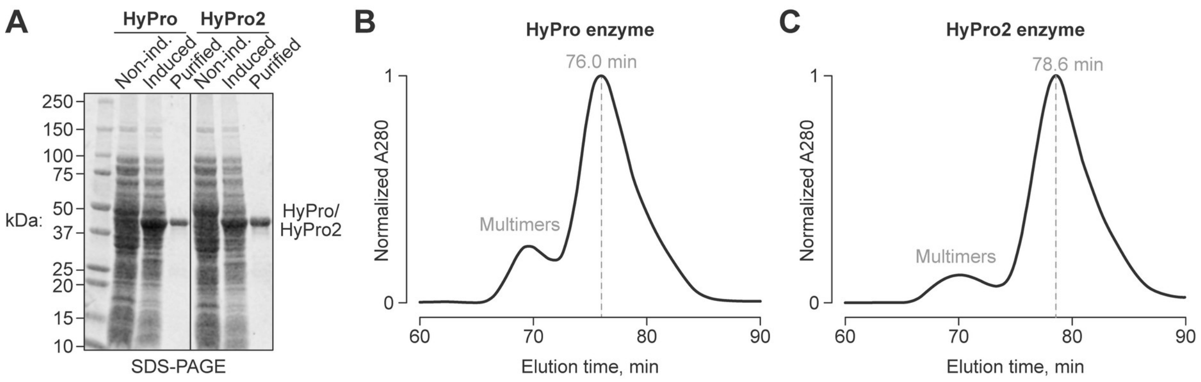

Supplementary Fig. 1: Expression and purification of HyPro and HyPro2 enzymes.

(A) SDS-PAGE analysis of SoluBL21 *E. coli* transformed with plasmids encoding HyPro or HyPro2, with and without IPTG induction. The gels also display HyPro and HyPro2 enzymes purified via metal affinity chromatography.

(B-C) Size-exclusion chromatography analysis suggests that, compared to (B) HyPro, (C) HyPro2 is a more compact protein less prone to multimerization. Note that we used only fractions from the main, non-multimerized peaks of the HyPro and HyPro2 enzyme for subsequent hybridization-proximity labeling experiments.

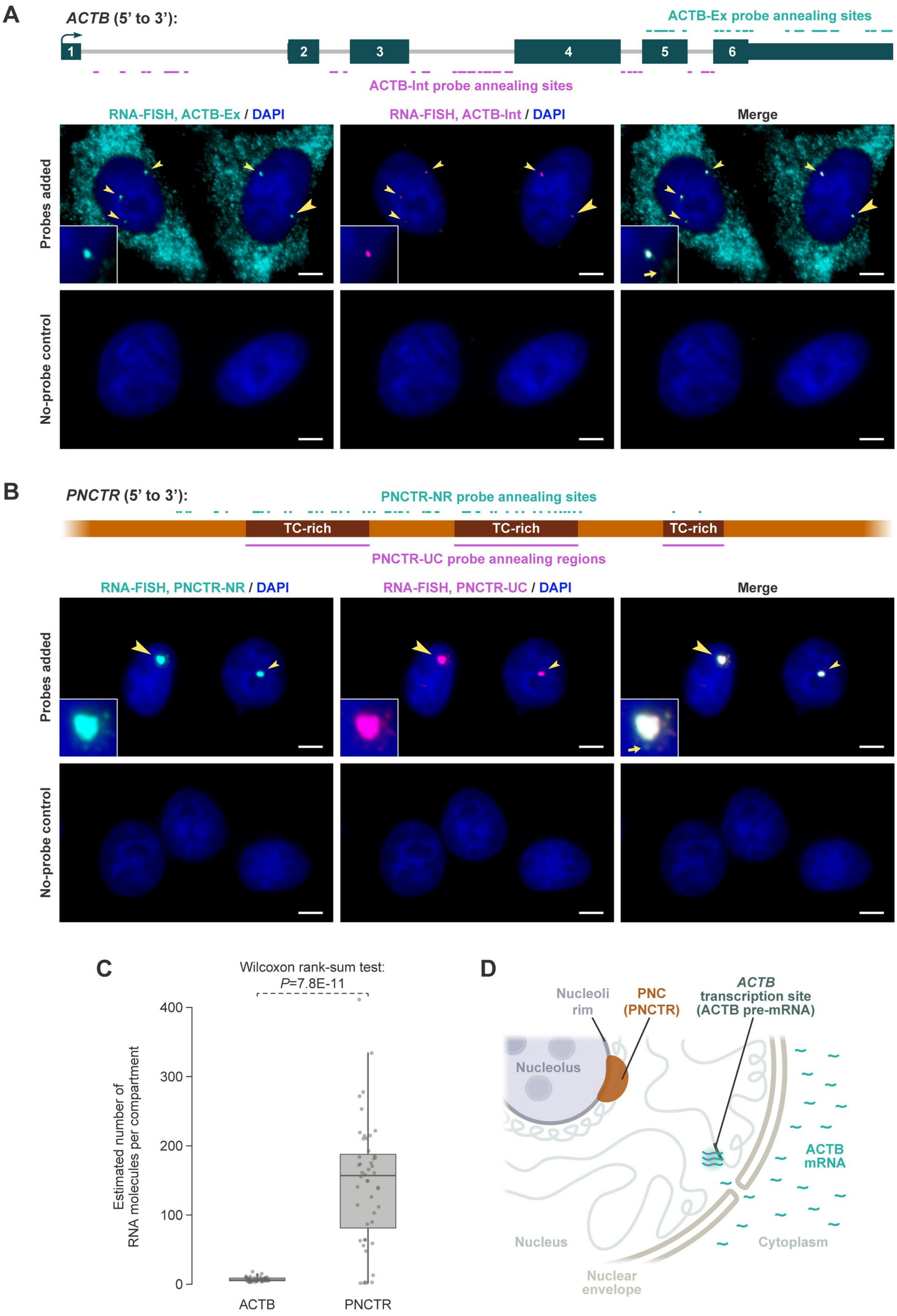

Supplementary Fig. 2: RNA-FISH analysis of PNCTR and ACTB transcripts.

(A) HeLa cells were co-stained with RNA-FISH probes targeting exonic (ACTB-Ex; cyan) or intronic sequences (ACTB-Int; magenta) of ACTB transcripts. The two signals colocalize in 2-3 nuclear foci corresponding to *ACTB* transcription sites (arrowheads; HeLa is quasi-triploid for chromosome 7 [1], which encodes *ACTB*). Mature ACTB mRNAs, containing only exons, are predominantly detected in the cytoplasm, as expected. The transcription site marked by the large arrowhead is magnified 3× in the close-up. In the close-up, the arrow indicates an individual exon-positive, intron-negative ACTB mRNA released from the transcription site. Main images, maximum-intensity Z-stacks; close-ups, individual optical sections. Scale bars, 5 µm. The *ACTB* gene and probe-annealing regions are illustrated at the top. Negative control RNA-FISH data, where the cells were incubated without probes, are shown at the bottom.

(B) HeLa cells were co-stained with RNA-FISH probes targeting either the non-repetitive (PNCTR-NR; cyan) or UC-rich simple repeated sequences (PNCTR-UC; magenta) regions of the long noncoding RNA PNCTR, which localizes to the perinucleolar compartment (PNC; arrowheads; [2, 3, 4]). The PNC marked by the large arrowhead is magnified 3× in the close-up. In the close-up, the arrow indicates an individual PNCTR transcript diffusing away from the main PNC density. Main images, maximum-intensity Z-stacks; close-ups, individual optical sections. Scale bars, 5 µm. The PNCTR locus and probe-annealing regions are illustrated at the top. Negative control RNA-FISH data, where the cells were incubated without probes, are shown at the bottom.

(C) Numbers of RNA molecules per PNC and *ACTB* transcription site were estimated by FISH-quant (see Methods). The *ACTB* transcription site is a significantly smaller compartment compared to the PNC, comprising a median of only 6 ACTB pre-mRNAs. The data are from 3 RNA-FISH experiments, presented as a box plot, and compared by a two-sided Wilcoxon rank-sum test.

(D) Graphical summary of the data in (A-C).

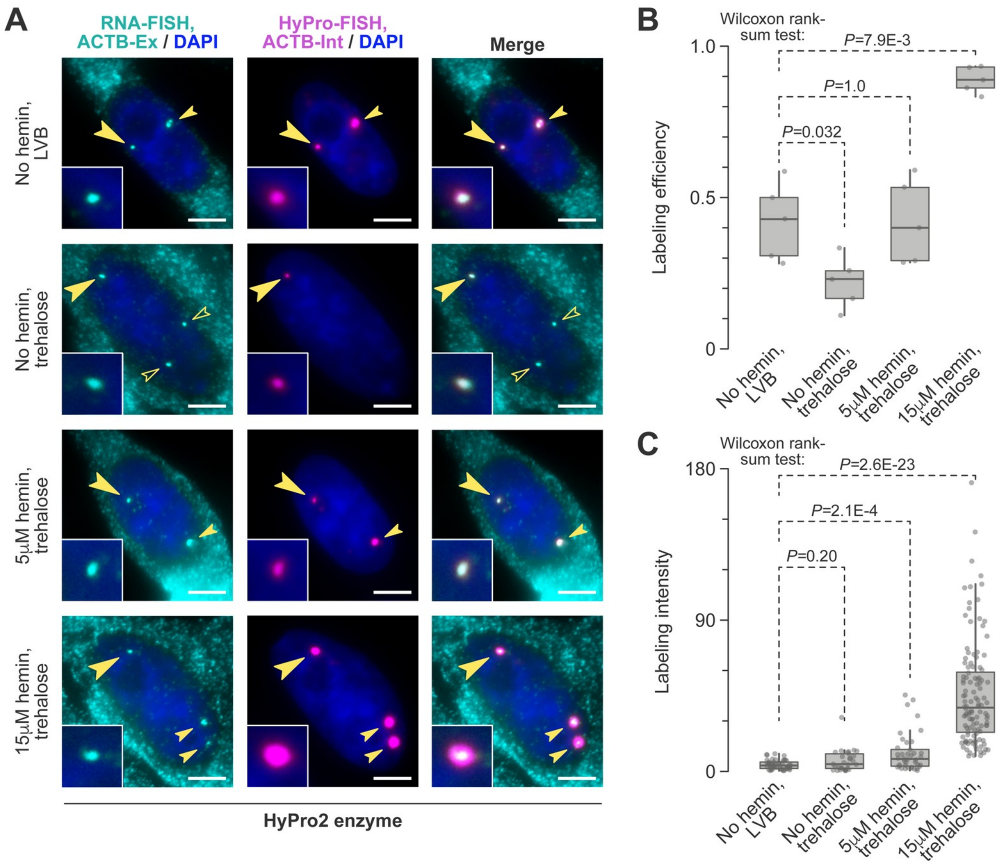

Supplementary Fig. 3: Testing the effect of hemin pre-incubation on proximity labeling. **(A)** Proximity labeling (magenta) of *ACTB* transcription sites by HyPro2 in LVB without hemin pre-incubation (row 1), in trehalose-containing buffer without hemin pre-incubation (row 2), or in trehalose-containing buffer with 5 µM or 15 µM hemin pre-incubation (rows 3 and 4). The samples were additionally co-stained by ACTB exon-specific RNA-FISH (cyan). Solid arrowheads, proximity-labeled transcription sites. Open arrowheads, transcription sites without proximity labeling. Transcription sites indicated by large arrowheads are magnified 3× in close-ups. Main images, maximum-intensity Z-stacks; close-ups, individual optical sections. Scale bars, 5 µm. All samples were imaged using identical microscopy settings. Note that pre-incubation with 5 µM hemin rescues the somewhat reduced labeling efficiency in the presence of trehalose, while 15 µM hemin oversaturates the signal.

(B-C) Box-plot quantification of (B) proximity-labeling efficiency (fraction of detectably biotinylated RNA-FISH-positive ACTB foci) and (C) the intensity of proximity-labeled RNA-FISH-positive foci in (A). Data in (B-C) were quantified from coverslip areas randomly selected from 3 labeling experiments, presented as box plots, and compared by a two-sided Wilcoxon rank-sum.

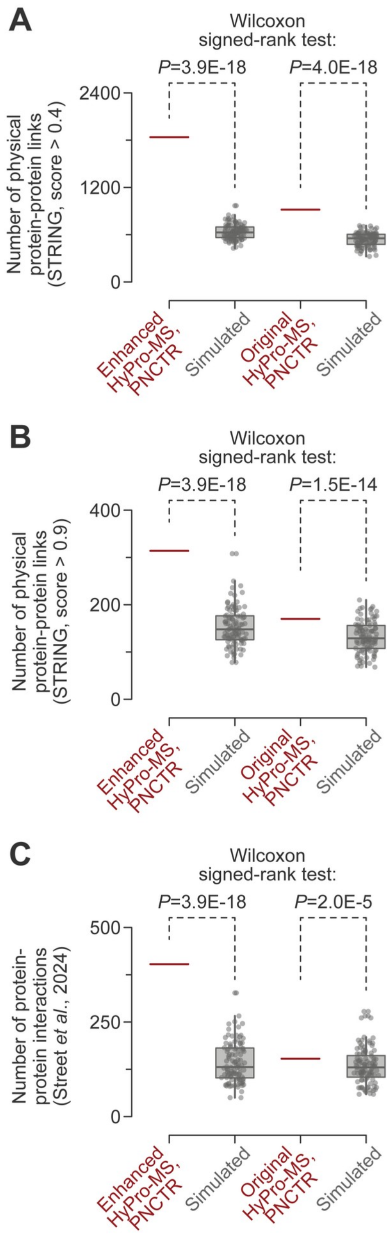

Supplementary Fig. 4: Enhanced HyPro-MS efficiently identifies RNA-associated protein complexes.

Compared to its original counterpart [3], the enhanced HyPro-MS procedure markedly improves the identification of physical protein-protein interactions within the PNCTR-associated proteome. This is supported by data from **(A-B)** the STRING database [5] and **(C)** the recently published RNA-aware protein-protein interactome resource [6]. In the STRING analyses, enhanced HyPro-MS consistently outperforms its predecessor, using either (A) the relaxed score > 0.4 cutoff or (B) the strict score > 0.9 cutoff. Similar results were observed with the intermediate score > 0.7 cutoff (Fig. 4D). In all cases, a two-sided Wilcoxon signed-rank test was used to compare the actual number of protein-protein interactions with those from simulated proteomes, in which the corresponding number of proteins was repeatedly sampled (n = 100) at random from the entire pool of mass spec-detectable nuclear proteins.

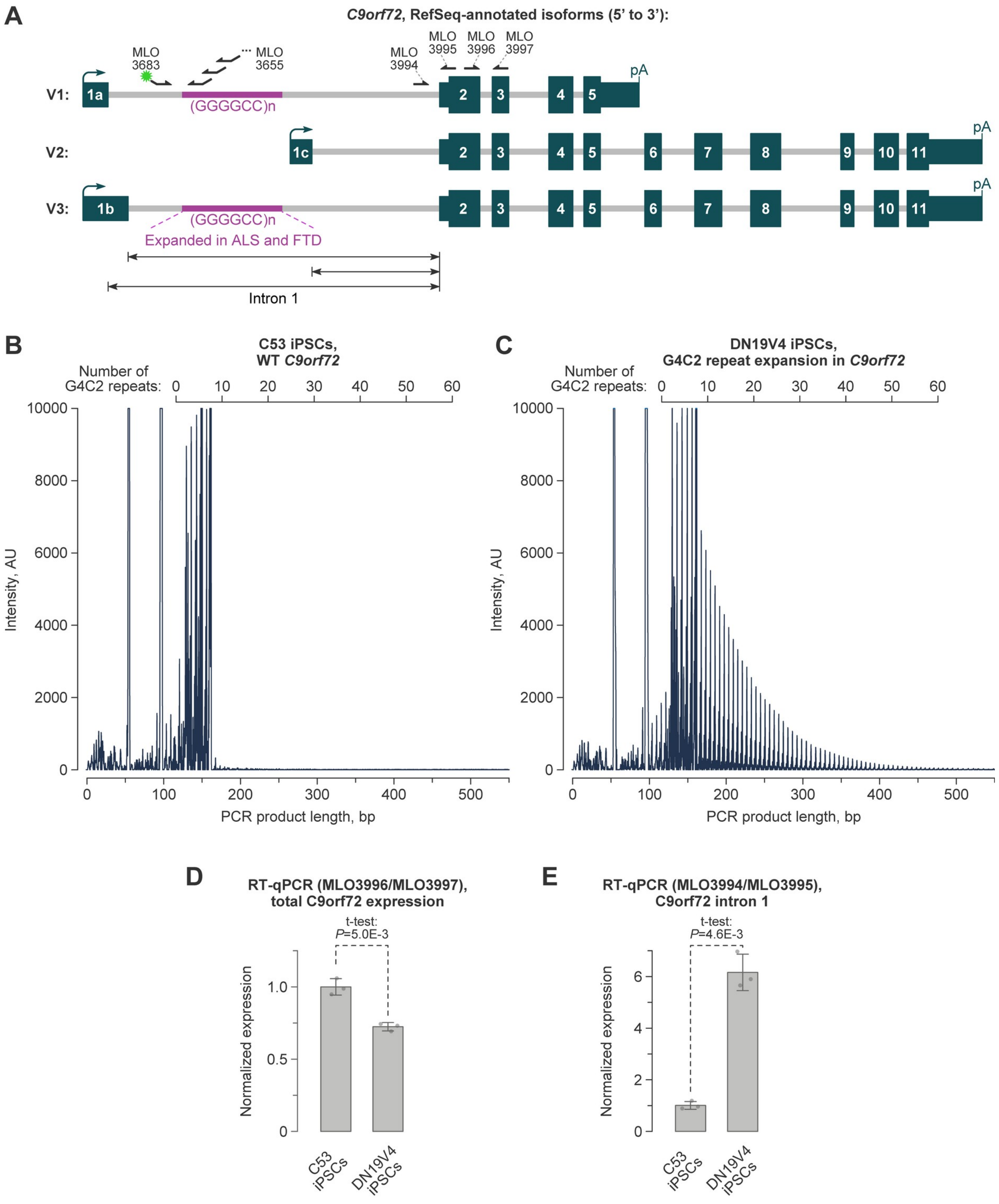

Supplementary Fig. 5: C9-ALS iPSCs with expanded G4C2 repeats accumulate C9orf72 transcripts with unspliced intron 1.

(A) Diagram of the C9orf72 gene and the primers used in this study.

(B-C) Genomic DNA was PCR-amplified using the MLO3655/MLO3683 primers introduced in (A). The results show that (B) both *C9orf72* alleles encode <10 copies of the G4C2 repeat in C53 iPSCs from a healthy donor. (C) Conversely, at least one *C9orf72* allele in C9-ALS

DN19V4 iPSCs contains an expanded G4C2 repeat.

(D-E) The expression of C9orf72 in iPSCs was analyzed by reverse transcription-quantitative PCR (RT-qPCR) with either (D) the MLO3996/MLO3997 primers, or (E) the MLO3994/MLO3995 primers, introduced in (A). Note that, although the overall *C9orf72* expression is somewhat lower in DN19V4 compared to C53, the abundance of C9orf72 transcripts retaining intron 1 is ~6-fold higher in DN19V4 than in C53. Data in (D-E) are from triplicated RT-qPCR assays, presented as mean ±SD and compared by a two-sided t-test, assuming unequal variance.

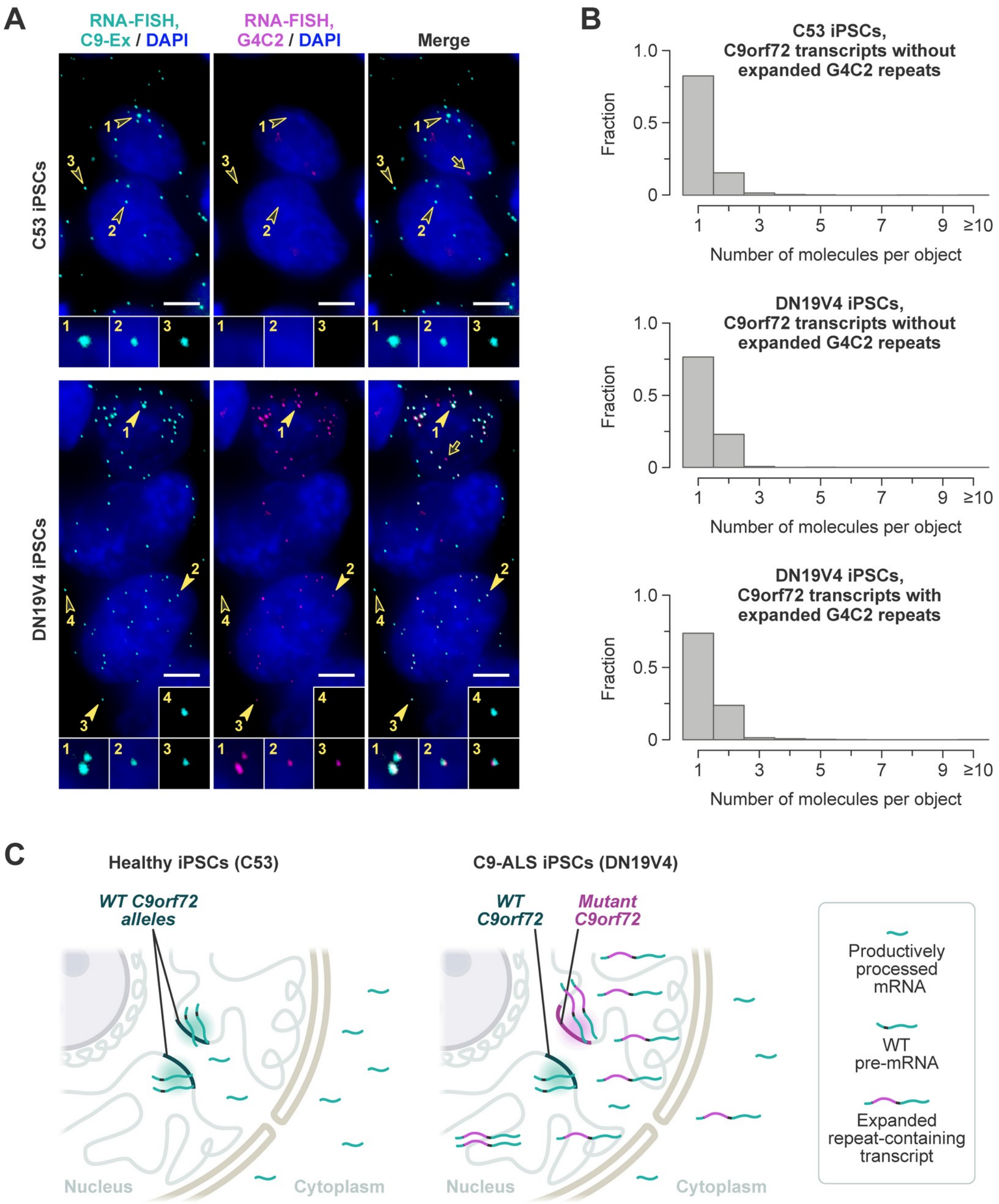

Supplementary Fig. 6: C9orf72 transcripts containing expanded G4C2 repeats tend to accumulate in C9-ALS iPSC nuclei as single-molecule foci.

(A) iPSCs were co-stained with RNA-FISH probes targeting C9orf72 exons (C9-Ex; cyan) and G4C2 repeats (G4C2; magenta). In wild-type C53 iPSCs, all C9-Ex-positive foci are G4C2-negative and well-distributed across the nucleus and cytoplasm (open arrowheads and the corresponding close-ups in the C53 iPSCs panels). In contrast, a substantial fraction of C9-Ex-positive foci in C9-ALS DN19V4 iPSCs are also G4C2-positive (solid arrowheads; corresponding close-ups provided). These double-positive foci predominantly localize to the nucleus, although some are occasionally observed in the cytoplasm. Interestingly, different

nuclei DN19V4 appear to express distinct numbers of double-positive foci, an effect that should be investigated in further studies. The C9-Ex-positive/G4C2-negative foci detected in DN19V4 iPSCs may originate from the wild-type *C9orf72* allele or represent productively spliced transcripts of the mutant *C9orf72* allele (open arrowheads and the corresponding close-ups in the DN19V4 iPSCs panels). Examples of nonspecific signal occasionally detected by the G4C2 probe in C53 and possibly DN19V4 cells are marked by open arrows. Main images, maximum-intensity Z-stacks; close-ups, individual optical sections. Scale bars, 5 µm.

(B) The number of RNA molecules per C9orf72 focus was estimated using FISH-quant based on the exon-probe data from (A). Notably, most RNA-FISH foci in C53 and DN19V4 iPSCs correspond to a single RNA molecule, with occasional instances of two, regardless of the presence or absence of the expanded G4C2 repeat.

(C) Graphical summary of the data in (A-B).

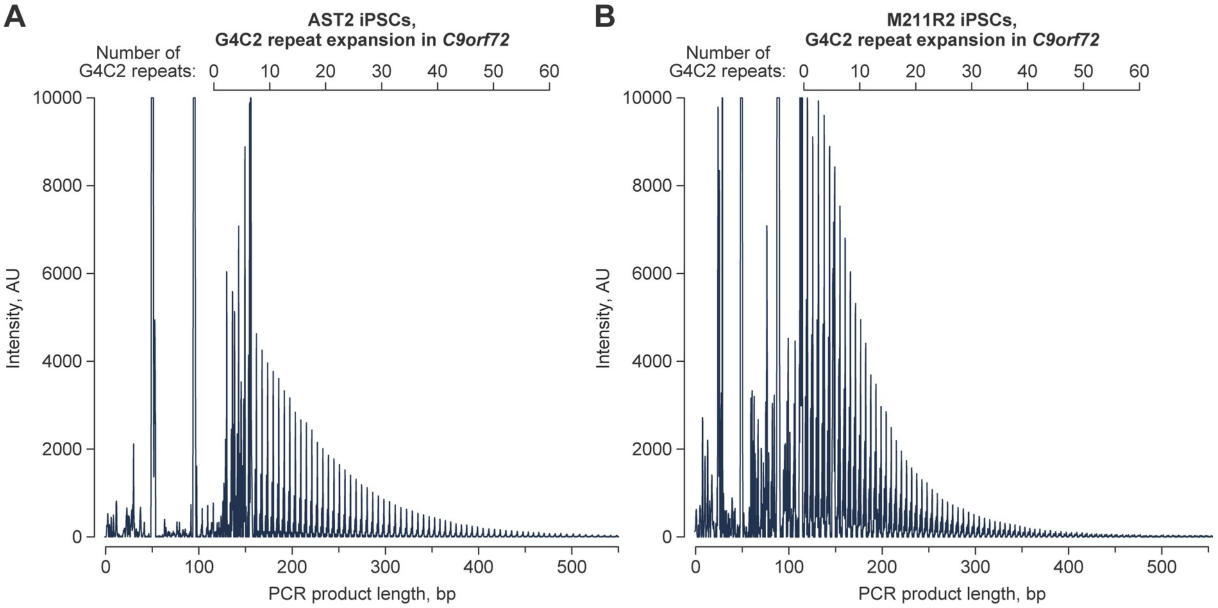

Supplementary Fig. 7: Repeat-prime PCR genotyping of AST2 and M211R2 lines C9-ALS iPSC lines.

Two additional C9-ALS iPSC lines, **(A)** AST2 and **(B)** M211R2, were analyzed by repeat-primed PCR [7] using the MLO3655/MLO3683 primers introduced in Supplementary Fig. 5A. The results confirm that at least one *C9orf72* allele in both AST2 and M211R2 lines contains an expanded G4C2 repeat, similar to the DN19V4 line in Supplementary Fig. 5C.

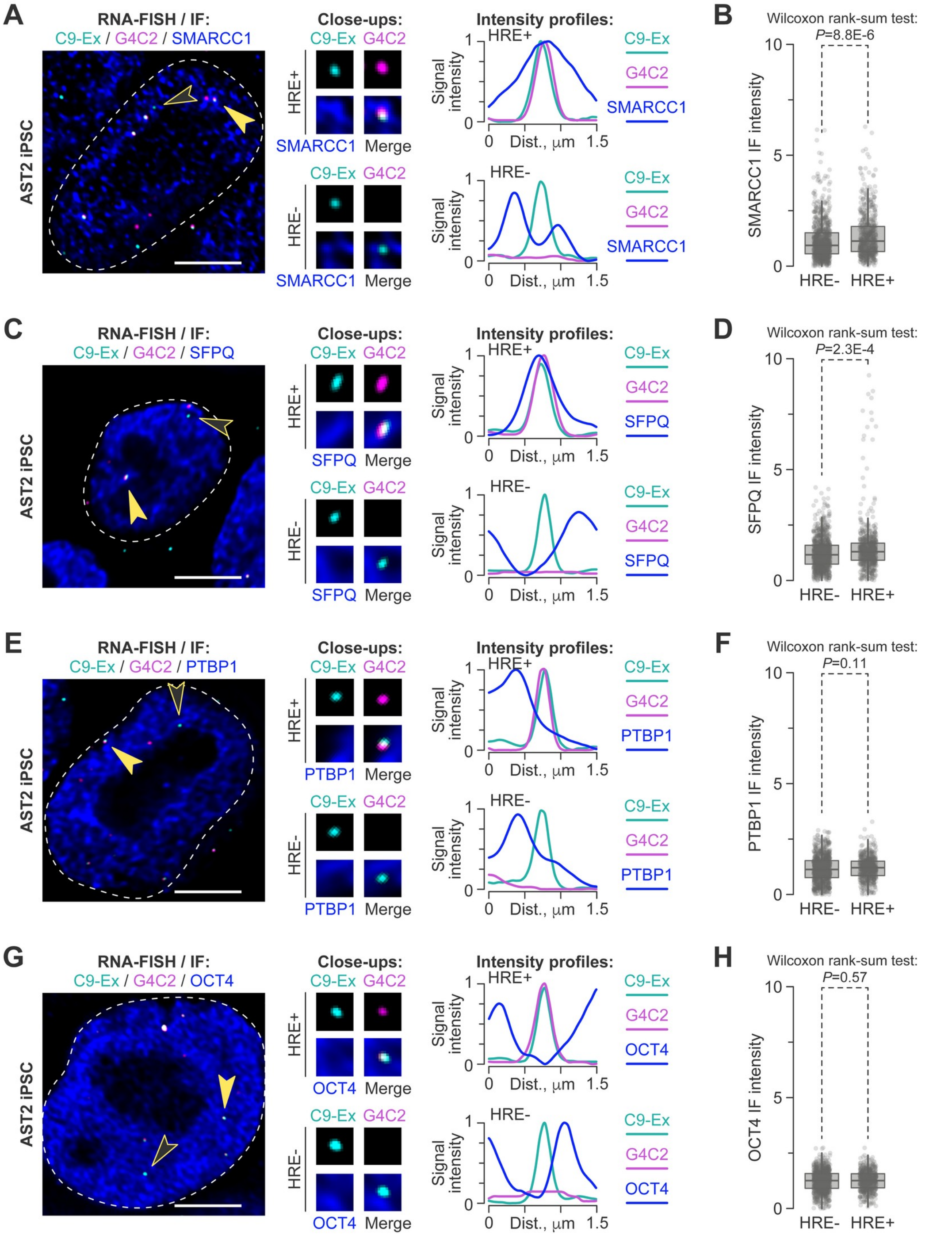

Supplementary Fig. 8: C9orf72 transcripts containing expanded G4C2 repeats colocalize with paraspeckle markers in AST2 C9-ALS iPSC line.

(A, C, E, G) AST2 iPSCs were co-stained with RNA-FISH probes targeting C9orf72 exons (C9-Ex; cyan) and G4C2 repeats (G4C2; magenta), along with antibodies (blue) against the paraspeckle markers (A) SMARCC1 or (C) SFPQ, or non-paraspeckle proteins enriched in iPSC nuclei (E) PTBP1 or (G) OCT4. Solid arrowheads, C9orf72 transcripts with G4C2 hexanucleotide repeat expansion (HRE+); open arrowheads, C9orf72 transcripts without G4C2 hexanucleotide repeat expansion (HRE-). The marked transcripts are magnified 3× in

the close-ups in the middle of each panel. Signal intensity profiles on the right are plotted along 1.5 µm virtual lines drawn in the direction indicated by the arrowheads in the main images. For each cell, signal intensities in each channel are normalized to a maximum value of 1. In (A) and (C), HRE+ transcripts tend to colocalize with paraspeckle marker-positive densities, whereas HRE- transcripts do not. The PTBP1 and OCT4 IF signals in (E) and (G) show no distinction between transcript types. Main images, deconvolved maximum-intensity Z-stacks; close-ups, deconvolved individual optical sections. Scale bars, 5 µm.

(B, D, F, H) Quantification of immunofluorescence signal intensity near C9orf72 transcripts with G4C2 hexanucleotide repeat expansion (HRE+) vs. C9orf72 transcripts without G4C2 hexanucleotide repeat expansion (HRE-). Paraspeckle marker staining intensity is significantly higher near HRE+ transcripts for (B) SMARCC1 and (D) SFPQ. No significant difference is observed for (F) PTBP1 and (H) OCT4 controls. Quantifications are from 3 staining experiments, presented as box plots, and compared by a two-sided Wilcoxon rank-sum test.

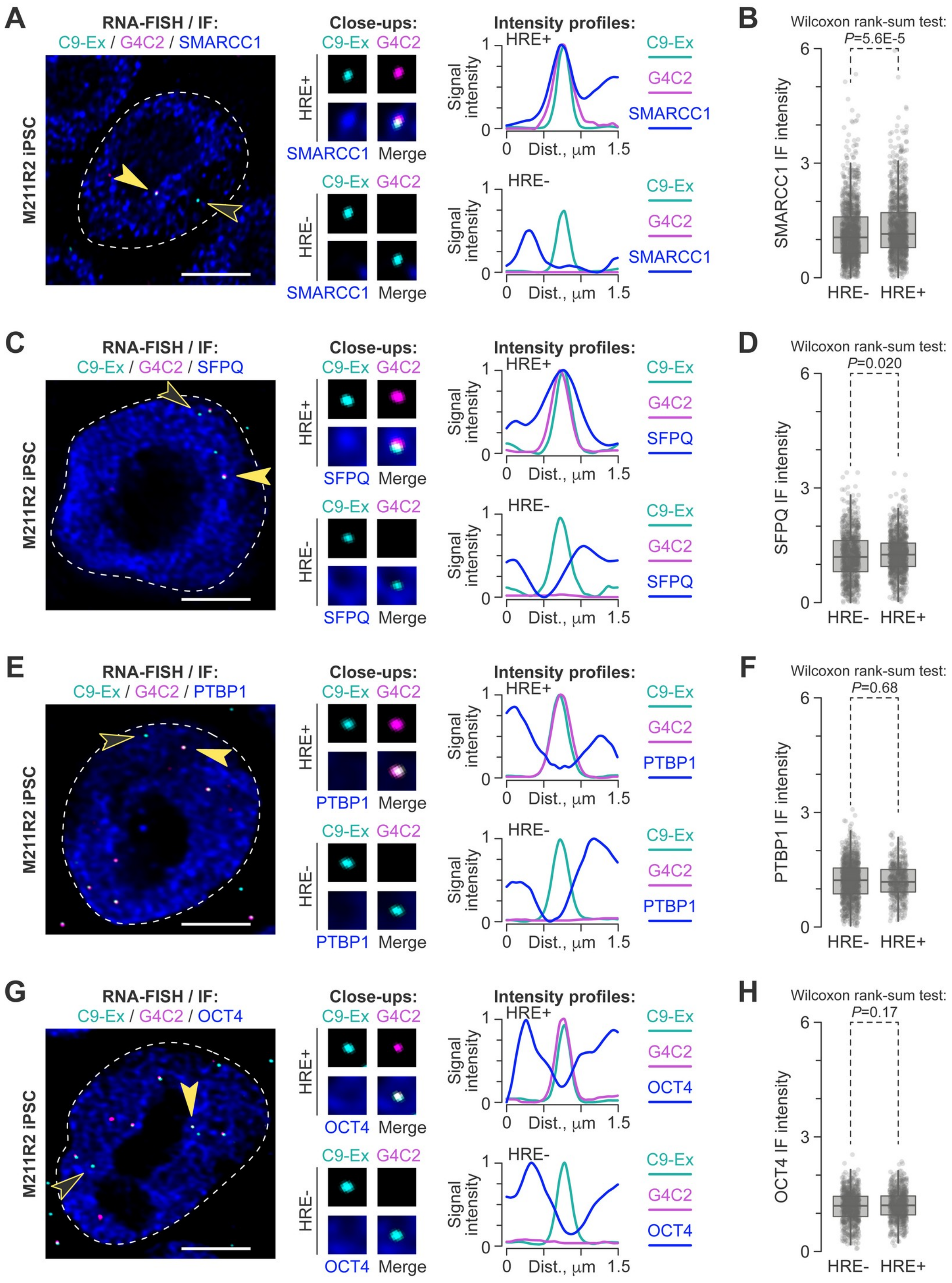

Supplementary Fig. 9: HRE+ C9orf72 transcripts colocalize with paraspeckle markers in M211R2 C9-ALS iPSC line.

(A, C, E, G) M211R2 iPSCs were co-stained with RNA-FISH probes targeting C9orf72 exons (C9-Ex; cyan) and G4C2 repeats (G4C2; magenta), along with antibodies (blue) against the paraspeckle markers (A) SMARCC1 or (C) SFPQ, or non-paraspeckle proteins enriched in iPSC nuclei (E) PTBP1 or (G) OCT4. Solid arrowheads, HRE+ C9orf72 transcripts; open arrowheads, HRE- C9orf72 transcripts. The marked transcripts are magnified 3× in the close-ups in the middle of each panel. Signal intensity profiles on the right are plotted along 1.5 µm

virtual lines drawn in the direction indicated by the arrowheads in the main images. For each cell, signal intensities in each channel are normalized to a maximum value of 1. In (A) and (C), HRE+ transcripts tend to colocalize with paraspeckle marker-positive densities, whereas HRE- transcripts do not. The PTBP1 and OCT4 IF signals in (E) and (G) show no distinction between transcript types. Main images, deconvolved maximum-intensity Z-stacks; close-ups, deconvolved individual optical sections. Scale bars, 5 μm.

(B, D, F, H) Quantification of immunofluorescence signal intensity near HRE+ vs. HRE- C9orf72 transcripts. Paraspeckle marker staining intensity is significantly higher near HRE+ transcripts for (B) SMARCC1 and (D) SFPQ. No significant difference is observed for (F) PTBP1 and (H) OCT4 controls. Quantifications are from 3 staining experiments, presented as box plots, and compared by a two-sided Wilcoxon rank-sum test.

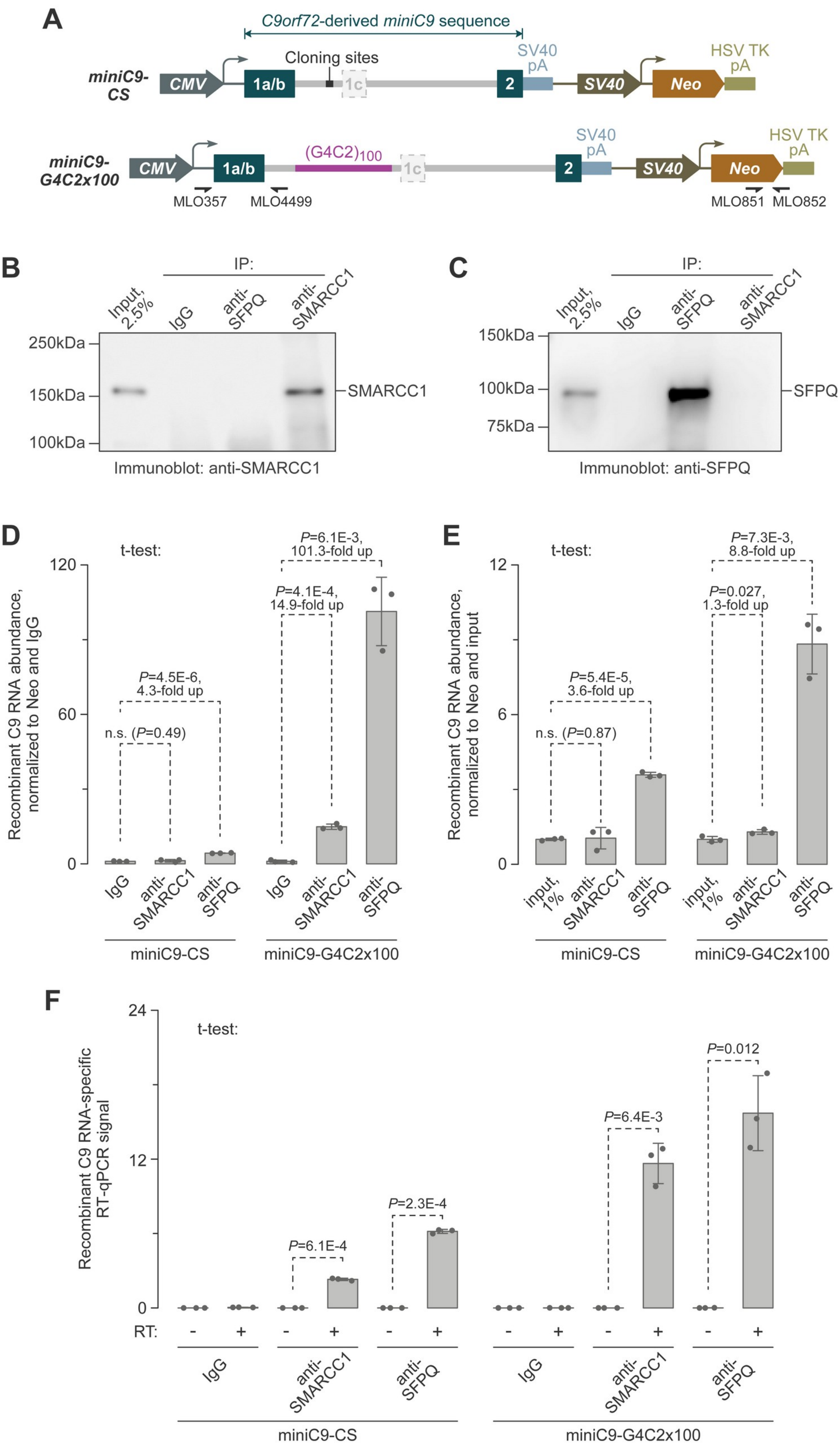

Supplementary Fig. 10: Formaldehyde-RNA immunoprecipitation confirms interactions between HRE+ C9orf72 transcripts and paraspeckle markers.

(A) *CMV* promoter-driven C9orf72 minigene *(C9mini)* constructs containing either 0 *(miniC9-*

CS) or 100 G4C2 repeats *(miniC9-G4C2x100).* Both constructs also include the *Neo* marker gene transcribed from the *SV40* promoter.

(B-C) Validation of anti-SMARCC1 and anti-SFPQ antibodies by immunoprecipitation from C53 iPSC lysates, followed by immunoblotting with (B) anti-SMARCC1 or (C) anti-SFPQ antibodies. Immunoprecipitation using non-immune IgG served as a negative control.

(D-F) C53 iPSCs transfected with either *miniC9-CS* or *miniC9-G4C2x100* constructs were treated with 1% formaldehyde, and interactions between miniC9 transcripts and SMARCC1 or SFPQ were assessed by immunoprecipitation with the respective antibodies, followed by RT-qPCR analysis. Non-immune IgG was used as a negative control. miniC9-specific RT-qPCR signals were normalized either to Neo transcript levels (using the ΔCt method) and the IgG control (D), or to Neo transcript levels and an input sample taken prior to immunoprecipitation (E). Both normalization methods confirm that miniC9-G4C2x100 transcripts interact more efficiently with SMARCC1 and SFPQ than miniC9-CS transcripts. (F) The inclusion of reverse transcriptase-negative (RT-) controls confirms the specificity of the assay in detecting RNA-protein interactions. In this case, RT-qPCR signals were ΔCt-normalized to minigene-specific Ct averages across all RT+ pull-down samples. Data in (D-F) are from triplicated formaldehyde-RNA immunoprecipitation/RT-qPCR assays, presented as mean ±SD and compared by a two-sided t-test, assuming unequal variance.

Supplementary References

1. Landry JJ*, et al.* The genomic and transcriptomic landscape of a HeLa cell line. *G3 (Bethesda)* **3**, 1213-1224 (2013).

2. Makeyev EV, Huang S. The perinucleolar compartment: structure, function, and utility in anti-cancer drug development. *Nucleus* **15**, 2306777 (2024).

3. Yap K, Chung TH, Makeyev EV. Hybridization-proximity labeling reveals spatially ordered interactions of nuclear RNA compartments. *Mol Cell* **82**, 463-478 e411 (2022).

4. Yap K, Mukhina S, Zhang G, Tan JSC, Ong HS, Makeyev EV. A Short Tandem Repeat-Enriched RNA Assembles a Nuclear Compartment to Control Alternative Splicing and Promote Cell Survival. *Mol Cell* **72**, 525-540 e513 (2018).

5. Szklarczyk D*, et al.* The STRING database in 2023: protein-protein association networks and functional enrichment analyses for any sequenced genome of interest. *Nucleic Acids Res* **51**, D638-D646 (2023).

6. Street LA*, et al.* Large-scale map of RNA-binding protein interactomes across the mRNA life cycle. *Mol Cell* **84**, 3790-3809 e3798 (2024).

7. DeJesus-Hernandez M*, et al.* Expanded GGGGCC hexanucleotide repeat in noncoding region of C9ORF72 causes chromosome 9p-linked FTD and ALS. *Neuron* **72**, 245-256 (2011).
