# Supplementary material for: Enhanced hybridization-proximity labeling discovers protein interactomes of single RNA molecules
Source: Nat Commun. 2025 Oct 20;16:9257. doi: 10.1038/s41467-025-64282-5 (PMC12537909; doi:10.1038/s41467-025-64282-5)
Supplement: Supplementary file 2 — Description of Additional Supplementary Information [file 41467_2025_64282_MOESM2_ESM.pdf]

Description of Additional Supplementary Files

File Name: Supplementary Data 1
Description: Antisense oligonucleotide probes used in this study.

File Name: Supplementary Data 2
Description: PNCTR-proximal proteome identified by enhanced HyPro-MS.
Proteins were shortlisted using the DEP package (Zhang X, Smits AH, van Tilburg GB, Ovaa H, Huber W, Vermeulen M. Nat Protoc 13, 530-550 (2018); https://bioconductor.org/packages/release/bioc/html/DEP.html), without adjustment of moderated t-test P-values for multiple testing. See Methods for details.

File Name: Supplementary Data 3
Description: Metascape analysis of proteins associated with PNCTR/PNC in HeLa cell nuclei.
Functional annotation of nuclear proteins identified by enhanced HyPro-MS was performed using Metascape (Zhou Y, et al. Nat Commun 10, 1523 (2019); https://metascape.org), incorporating GO Biological Processes, GO Cellular Components, and DisGeNET terms and maintaining QC as part of the enrichment calculations. Enrichment P-values were calculated using a one-sided hypergeometric test. Q-values are P-values adjusted for multiple testing using the Benjamini-Hochberg procedure.

File Name: Supplementary Data 4
Description: ACTB transcription-proximal proteome identified by enhanced HyPro-MS.
Proteins were shortlisted using the DEP package (Zhang X, Smits AH, van Tilburg GB, Ovaa H, Huber W, Vermeulen M. Nat Protoc 13, 530-550 (2018); https://bioconductor.org/packages/release/bioc/html/DEP.html), without adjustment of moderated t-test P-values for multiple testing. See Methods for details.

File Name: Supplementary Data 5
Description: Metascape analysis of proteins associated with ACTB transcription sites in HeLa cell nuclei.
Functional annotation of nuclear proteins identified by enhanced HyPro-MS was performed using Metascape (Zhou Y, et al. Nat Commun 10, 1523 (2019); https://metascape.org), incorporating GO Biological Processes, GO Cellular Components, and DisGeNET terms and maintaining QC as part of the enrichment calculations. Enrichment P-values were calculated using a one-sided hypergeometric test. Q-values are P-values adjusted for multiple testing using the Benjamini-Hochberg procedure.

File Name: Supplementary Data 6
Description: Proteins associated with mutant C9orf72 transcripts in C9-ALS iPSCs.
Proteins were shortlisted using the DEP package (Zhang X, Smits AH, van Tilburg GB, Ovaa H, Huber W, Vermeulen M. Nat Protoc 13, 530-550 (2018); https://bioconductor.org/packages/release/bioc/html/DEP.html), without adjustment of moderated t-test P-values for multiple testing. See Methods for details.

File Name: Supplementary Data 7
Description: Proteins consistently interacting with synthetic G4C2 repeat-containing transcripts in published studies.

File Name: Supplementary Data 8
Description: Metascape analysis of proteins associated with mutant C9orf72 transcripts in C9-ALS iPSC nuclei. Functional annotation of nuclear proteins identified by enhanced HyPro-MS was performed using Metascape (Zhou Y, et al. Nat Commun 10, 1523 (2019); https://metascape.org), incorporating GO Biological Processes, GO Cellular Components, and DisGeNET terms and maintaining QC as part of the enrichment calculations. Enrichment P-values were calculated using a one-sided hypergeometric test. Q-values are P-values adjusted for multiple testing using the Benjamini-Hochberg procedure.
