# Supplementary material for: Enhanced hybridization-proximity labeling discovers protein interactomes of single RNA molecules
Source: Nat Commun. 2025 Oct 20;16:9257. doi: 10.1038/s41467-025-64282-5 (PMC12537909; doi:10.1038/s41467-025-64282-5)
Supplement: Supplementary file 11 — Reporting Summary [file 41467_2025_64282_MOESM11_ESM.pdf]

nature portfolio

Corresponding author(s): Eugene Makeyev

Last updated by author(s): Aug 20, 2025

Reporting Summary

Nature Portfolio wishes to improve the reproducibility of the work that we publish. This form provides structure for consistency and transparency in reporting. For further information on Nature Portfolio policies, see our Editorial Policies and the Editorial Policy Checklist.

Statistics

For all statistical analyses, confirm that the following items are present in the figure legend, table legend, main text, or Methods section.

n/a	Confirmed	
☐	☒	The exact sample size (*n*) for each experimental group/condition, given as a discrete number and unit of measurement
☐	☒	A statement on whether measurements were taken from distinct samples or whether the same sample was measured repeatedly
☐	☒	The statistical test(s) used AND whether they are one- or two-sided<br>*Only common tests should be described solely by name; describe more complex techniques in the Methods section.*
☒	☐	A description of all covariates tested
☐	☒	A description of any assumptions or corrections, such as tests of normality and adjustment for multiple comparisons
☐	☒	A full description of the statistical parameters including central tendency (e.g. means) or other basic estimates (e.g. regression coefficient) AND variation (e.g. standard deviation) or associated estimates of uncertainty (e.g. confidence intervals)
☐	☒	For null hypothesis testing, the test statistic (e.g. *F*, *t*, *r*) with confidence intervals, effect sizes, degrees of freedom and *P* value noted<br>*Give P values as exact values whenever suitable.*
☒	☐	For Bayesian analysis, information on the choice of priors and Markov chain Monte Carlo settings
☒	☐	For hierarchical and complex designs, identification of the appropriate level for tests and full reporting of outcomes
☒	☐	Estimates of effect sizes (e.g. Cohen's *d*, Pearson's *r*), indicating how they were calculated

Our web collection on statistics for biologists contains articles on many of the points above.

Software and code

Policy information about availability of computer code

Data collection	ZEISS ZEN 2.5 Pro (https://www.zeiss.com/microscopy/en/products/software/zeiss-zen.html)
Data analysis	R, v4.4.3 (https://www.r-project.org/)<br>Python, v3.8.13 (https://www.python.org/)<br>FIJI, v1.53t (https://imagej.net/software/fiji/)<br>Cellpose, v2.2.3 (https://pypi.org/project/cellpose/)<br>FISH-quant, v2 (https://fish-quant.github.io/)<br>LightCycler 96 software, v1.1.0.1320 (https://lifescience.roche.com)<br>Image Studio Software, v6.0 (https://www.licorbio.com/image-studio)<br>Peak Scanner 2 (https://www.thermofisher.com/)<br>Proteome Discoverer, v2.2 (Thermo Fisher Scientific, UK)<br>Mascot, v2.6.0 (www.matrixscience.com)<br>DEP, v1.26.0 (https://bioconductor.org/packages/release/bioc/html/DEP.html)<br>Metascape, v3.5 (https://metascape.org)<br>In-house image analysis scripts (https://doi.org/10.6084/m9.figshare.29282240)

For manuscripts utilizing custom algorithms or software that are central to the research but not yet described in published literature, software must be made available to editors and reviewers. We strongly encourage code deposition in a community repository (e.g. GitHub). See the Nature Portfolio guidelines for submitting code & software for further information.

Data

Policy information about availability of data

All manuscripts must include a data availability statement. This statement should provide the following information, where applicable:

- Accession codes, unique identifiers, or web links for publicly available datasets
- A description of any restrictions on data availability
- For clinical datasets or third party data, please ensure that the statement adheres to our policy

The proteomics data generated in this study have been deposited to PRIDE (https://www.ebi.ac.uk/pride) as projects PXD063191 (https://www.ebi.ac.uk/pride/archive/projects/PXD063191) and PXD063192 (https://www.ebi.ac.uk/pride/archive/projects/PXD063192). Uncropped microscopy images have been uploaded to figshare (https://doi.org/10.6084/m9.figshare.29282240). Other types of source data are provided with this paper in the Source Data file.

Research involving human participants, their data, or biological material

Policy information about studies with human participants or human data. See also policy information about sex, gender (identity/presentation), and sexual orientation and race, ethnicity and racism.

Reporting on sex and gender	We investigated HeLa cells (of female origin), as well as human induced pluripotent stem cell (iPSC) lines derived from male C9-ALS patients and a healthy male donor. Analyses based on sex, gender, or social groupings were not performed due to the limited number of cell lines included in our study. The primary inclusion criterion was the availability of well-characterized cell lines, as established in previous studies, including those conducted by our team
Reporting on race, ethnicity, or other socially relevant groupings	See above
Population characteristics	Not applicable
Recruitment	Not applicable
Ethics oversight	UK National Research Ethics Service and King's College London

Note that full information on the approval of the study protocol must also be provided in the manuscript.

Field-specific reporting

Please select the one below that is the best fit for your research. If you are not sure, read the appropriate sections before making your selection.

☒ Life sciences ☐ Behavioural & social sciences ☐ Ecological, evolutionary & environmental sciences

For a reference copy of the document with all sections, see nature.com/documents/nr-reporting-summary-flat.pdf

Life sciences study design

All studies must disclose on these points even when the disclosure is negative.

Sample size	No sample size calculations were performed. Experiments were typically conducted in triplicate, in accordance with standard practice in molecular and cell biology research.
Data exclusions	No data were excluded, except for experiments that failed due to technical reasons.
Replication	Data reproducibility was confirmed by performing at least two, and typically three or more, independent experiments. Experiments that failed due to technical reasons were not analyzed. All technically successful experiments were used for data analyses using appropriate statistical approaches. All technically sound attempts at replication were successful.
Randomization	Randomization was not considered since we worked only with human cell lines, laboratory strains of E.coli, synthetic reagents (e.g. primers) and commercial research products.
Blinding	Blinding was not required in our molecular biology work relying on quantifiable and objective data. In cell imaging experiments, bias was minimized by randomly selecting microscopy fields and automating data analysis using appropriate macros.

Reporting for specific materials, systems and methods

We require information from authors about some types of materials, experimental systems and methods used in many studies. Here, indicate whether each material, system or method listed is relevant to your study. If you are not sure if a list item applies to your research, read the appropriate section before selecting a response.

Materials & experimental systems

n/a	Involved in the study	
☐	☒	Antibodies
☐	☒	Eukaryotic cell lines
☒	☐	Palaeontology and archaeology
☒	☐	Animals and other organisms
☒	☐	Clinical data
☒	☐	Dual use research of concern
☒	☐	Plants

Methods

n/a	Involved in the study	
☒	☐	ChIP-seq
☒	☐	Flow cytometry
☒	☐	MRI-based neuroimaging

Antibodies

Antibodies used

Rabbit anti-OCT4 (Abcam, cat# ab19857; RRID: AB_445175); rabbit anti-FUS (Proteintech, cat# 11570-1-AP; RRID:AB_2247082); rabbit anti-PTBP1 (Abcam, cat# ab133734; RRID:AB_2814646); rabbit anti-SMARCC1 (Abcam, cat# ab22355; RRID:AB_2191988); rabbit anti-SMARCC1 (Abcam, cat# ab172638; RRID: AB_3697426); and rabbit anti-SFPQ (Abcam, cat# 38148; RRID:AB_945424)

Validation

Rabbit anti-OCT4: validation images and published applications can be accessed at https://www.abcam.com/en-us/products/primary-antibodies/oct4-antibody-ab19857

Rabbit anti-FUS: validation images and published applications can be accessed at https://www.ptglab.com/Products/FUS-Antibody-11570-1-AP.htm

Rabbit anti-PTBP1: validation images and published applications can be accessed at https://www.abcam.com/en-us/products/primary-antibodies/ptbp1-antibody-epr9048b-ab133734

Rabbit anti-SMARCC1 (Abcam, cat# ab22355): validation images and other details can be accessed at https://doc.abcam.com/legacy-unpublished/datasheets/com/datasheet_22355.pdf

Rabbit anti-SMARCC1 (Abcam, cat# ab172638): specificity tested in Fig. S10; additional validation images and published applications can be accessed at https://www.abcam.com/en-us/products/primary-antibodies/smarcc1-baf155-antibody-epr12395-chip-grade-ab172638

Rabbit anti-SFPQ: specificity tested in Fig. S10; additional validation images and published applications can be accessed at https://www.abcam.com/en-us/products/primary-antibodies/sfpq-antibody-ab38148

Eukaryotic cell lines

Policy information about cell lines and Sex and Gender in Research

Cell line source(s)

HeLa (ATCC CCL-2; female); C9-ALS male iPSC lines DN19V4, AST2, and M211R2 and the healthy male iPSC line C53 (derived and characterized by Siddharthan Chandran's, Agnes Nishimura's, Chris Shaw's, and Selina Wray's groups; PMIDs: 29367641 and 38507480)

Authentication

HeLa cells were authenticated based on their characteristic morphology (https://www.atcc.org/products/ccl-2#detailed-product-images) and the high incidence of the perinucleolar compartment (PMID: 20182614). iPSC lines were genotyped using the repeat-primed PCR approach (PMIDs: 21944778 and 29367641)

Mycoplasma contamination

All cell lines tested negative form mycoplasma contamination

Commonly misidentified lines (See ICLAC register)

Not applicable

Plants

Seed stocks

Not applicable

Novel plant genotypes

Not applicable

Authentication

Not applicable
